# Use of antimicrobial peptides as a feed additive for juvenile goats

**DOI:** 10.1038/s41598-017-12394-4

**Published:** 2017-09-25

**Authors:** Qi Liu, Shuhua Yao, Yun Chen, Shuang Gao, Yanyi Yang, Junliang Deng, Zhihua Ren, Liuhong Shen, Hengmin Cui, Yanchun Hu, Xiaoping Ma, Shumin Yu

**Affiliations:** The Key Laboratory of Animal Disease and Human Health of Sichuan Province, Key Laboratory of Environmental Hazard and Animal Disease of Sichuan Province, College of Veterinary Medicine, Sichuang Agricultural University, Chengdu Sichuang, 611130 China

## Abstract

Although antimicrobial peptides (AMPs) have been used as feed additives, only a few studies have examined their use in ruminants. In this study, we evaluated the use of AMPs(recombinant swine defensin and a fly antibacterial peptide were mixed by 1:1) as a medicated feed additive for juvenile goats. Dietary treatments included control groups (group I: 300 g concentrate; group III: 600 g concentrate), and AMP-supplemented groups (group II: 300 g concentrate + 3.0 g AMPs; group IV: 600 g concentrate + 3.0 g AMPs). AMP-treated groups exhibited an increase in bacterial genera, including *Fibrobacter*, *Anaerovibrio*, and *Succiniclasticum*, and the ciliate genus *Ophryoscolex*; as well a reduction in bacterial genera, such as *Selenomonas*, *Succinivibrio*, and *Treponema*, and the ciliate genera *Polyplastron*, *Entodinium*, and *Isotricha*. The changes in *Fibrobacter*, *Anaerovibrio*, *Ophryoscolex*, *Polyplastron*, *Entodinium*, and *Isotricha* were related to the concentrate. AMP treatment led to increased body weight, average daily weight gain, enzymatic activity (pectinase, xylanase, and lipase), especially in the normal concentrate group, and influence on ruminal fermentation function. In addition, goats treated with AMPs had higher rumen microorganism diversity indices than the control groups. Our results demonstrate that AMPs can be utilized as feed additives for juvenile goats.

## Introduction

The microbial environment in the rumen is quite complex and dynamic; this is due to several factors including type of diet^1,2^. The microbial community consists of bacteria (10^10^–10^11^ cells/mL), methanogenic archaea (10^7^–10^9^ cells/mL), ciliate protozoa (10^4^–10^6^ cells/mL), anaerobic fungi (10^3^–10^6^ cells/mL), and bacteriophages (10^9^–10^10^ particles/mL) present^3^. A major function of the microbiome is to ferment plant materials that can be ingested by ruminant animals^4–6^. Rumen regulation is one of the most important methods for improving feed efficiency, ruminant health, and ruminant livestock production performance. Several antibiotic compounds, such as monensin, hainanmycin, and virginiamycin, have been used to improve ruminal fermentation and the efficiency of nutrient utilization^7–9^. However, the overuse of antibiotics has raised concerns regarding product safety and environmental health, therefore, the use of antibiotics as animal feed additives has been banned in the European Union (European Union, 2003).

Antimicrobial peptides (AMPs) are widespread in bacteria, animals, and plants and provide opportunities for novel research. In addition to antimicrobial properties^10^, previous studies have demonstrated antifungal^11^, antiviral^12^, anti-parasitic^13^, and antitumor activities^14^. AMP-induced immunoregulatory and antioxidant activities have been shown to be mediated by cationic charge, amphipathicity, amino acid composition, and structure^15^. AMPs have also been demonstrated to improve performance, nutrient retention, and intestinal morphology, and to reduce the incidence of diarrhoea in livestock animals^16–19^. Peng *et al*.^20^ demonstrated that dietary supplementation with crude rpBD2 (recombinant porcine β-defensin 2) has beneficial effects on growth and intestinal morphology of weaned piglets, reducing the incidence of post-weaning diarrhoea and the numbers of potential pathogens in the caecum. AMPs could therefore serve as potential alternatives to antibiotics in livestock production. However, there is insufficient information on the effects of AMPs on rumen digestion, as only a limited number of inconclusive studies have examined the use of AMPs as alternatives to feed antibiotics and growth promoters in ruminant nutrition. Previous studies in our laboratory have shown that adding AMPs (composed of recombinant swine defensin and a fly antibacterial peptide at a blending ratio of 50:50) in feed can improve growth and immunity of weaned piglets^15^. Based on our previous findings and the reported bactericidal effects of AMPs, we hypothesized that dietary AMP supplementation could affect rumen microbiology, and therefore ruminal fermentation. In the present study, we investigated the effects of AMPs on rumen fermentation function and rumen microbial community structure in Chuanzhong black goats.

## Results

### Growth performance

**Figure 1:**
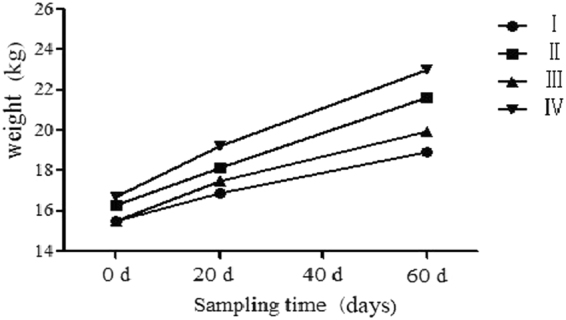
Weight changes in goats per group.

The mean initial body weights in groups I, II, III, and IV were 15.54 kg, 15.51 kg, 16.31 kg, and 16.70 kg, respectively. The weights increased to 18.96 kg, 19.93 kg, 21.60 kg, and 22.99 kg, respectively, following 60 days of experimental feeding (Table 1 and Fig. 1). The average daily gain (g) was significantly higher (*P* < *0.05*) in the AMP-supplemented groups (II, IV) than in the control groups (I, III; Table 2).

**Table 1:** Changes in goat body weight and average daily gain.

Item	Time point(day)/Time range	Groups				*P-Value*			
		I	II	III	IV	I VS II	III VS IV	I VS III	II VS IV
weight (kg)	0d	15.54 ± 0.21	15.51 ± 0.84	16.31 ± 1.96	16.70 ± 0.97	0.890	0.698	0.465	0.067
	20d	16.91 ± 0.18	17.49 ± 0.59	18.13 ± 0.49	19.23 ± 0.61	0.167	0.099	0.011^a^	0.024^a^
	60d	18.96 ± 0.19	19.93 ± 0.18	21.60 ± 0.77	22.99 ± 0.72	0.003^a^	0.094	0.005^a^	0.004^a^
average daily gain (g/d)	0d-20d	68.50 ± 7.45	90.63 ± 3.15	99.88 ± 5.81	126.26 ± 37.50	0.024^a^	0.280	0.010^a^	0.163
	20d-60d	51.38 ± 6.19	86.88 ± 8.26	61.06 ± 5.72	94.06 ± 19.04	0.002^a^	0.033^a^	0.197	0.639
	0d-60d	57.08 ± 1.89	88.12 ± 6.25	74.00 ± 3.37	104.79 ± 13.55	0.002^a^	0.009^a^	0.007^a^	0.168

^a^Mean significant difference (*P* < 0.05).

**Table 2:** Changes in ruminal fermentation parameters in goat rumen fluid.

Parameter		Groups				*P-Value*			
		I	II	III	IV	I VS II	III VS IV	I VS III	II VS IV
pH	0d	6.89 ± 0.03	6.88 ± 0.03	6.88 ± 0.03	6.87 ± 0.04	0.215	0.893	0.504	0.714
	20d	6.87 ± 0.05	6.82 ± 0.04	6.78 ± 0.04	6.74 ± 0.01	0.48	0.054	0.006^a^	0.119
	60d	6.95 ± 0.01	6.92 ± 0.06	6.85 ± 0.27	6.79 ± 0.03	0.49	0.659	0.515	0.024
Ammonia (mg/100 mL)	0d	11.19 ± 0.21	11.19 ± 0.36	11.01 ± 0.24	11.06 ± 0.22	0.99	0.067	0.256	0.334
	20d	9.55 ± 0.29	8.86 ± 0.31	12.53 ± 0.42	11.95 ± 0.37	0.016^a^	0.189	0.003^a^	0.003^a^
	60d	10.32 ± 0.15	9.64 ± 0.33	17.79 ± 0.86	16.49 ± 0.51	0.008^a^	0.108	0.001^a^	0.001^a^
T-VFA (mmol/L)	0d	75.59 ± 1.27	75.48 ± 0.89	75.28 ± 1.39	75.33 ± 1.18	0.801	0.916	0.591	0.734
	20d	69.22 ± 1.25	88.83 ± 2.13	86.97 ± 2.27	72.67 ± 2.23	0.001^a^	0.001^a^	0.001^a^	0.002^a^
	60d	63.97 ± 1.52	68.83 ± 1.85	69.03 ± 2.20	62.57 ± 1.68	0.06	0.007^a^	0.001^a^	0.043^a^
Acetate (mmol/l)	0d	51.70 ± 1.60	51.61 ± 0.91	51.46 ± 1.59	51.31 ± 1.52	0.849	0.76	0.73	0.719
	20d	46.21 ± 1.50	61.12 ± 1.99	61.52 ± 2.25	48.58 ± 1.75	0.004^a^	0.001^a^	0.001^a^	0.002^a^
	60d	41.94 ± 1.36	45.51 ± 2.07	46.91 ± 1.78	40.14 ± 1.54	0.096	0.002^a^	0.003^a^	0.064
Propionate (mmol/l)	0d	15.30 ± 0.40	15.27 ± 0.20	15.22 ± 0.34	15.41 ± 0.41	0.909	0.354	0.671	0.573
	20d	14.38 ± 0.48	19.05 ± 0.85	15.26 ± 0.71	15.47 ± 0.88	0.003^a^	0.52	0.003^a^	0.02^a^
	60d	13.24 ± 0.47	14.43 ± 0.73	12.88 ± 0.58	13.46 ± 0.60	0.099	0.374	0.523	0.039^a^
Butyrate (mmol/l)	0d	8.60 ± 0.15	8.59 ± 0.14	8.60 ± 0.19	8.61 ± 0.23	0.945	0.782	0.885	0.926
	20d	8.64 ± 0.44	8.67 ± 0.30	10.20 ± 0.58	8.61 ± 0.53	0.838	0.021^a^	0.033^a^	0.863
	60d	8.79 ± 0.34	8.88 ± 0.52	9.24 ± 0.22	8.98 ± 0.17	0.507	0.124	0.108	0.711
Acetate + Butyrate to Propionate ratio	0d	3.95 ± 0.20	3.94 ± 0.09	3.95 ± 0.17	3.89 ± 0.19	0.906	0.195	0.973	0.634
	20d	3.82 ± 0.17	3.67 ± 0.24	4.71 ± 0.26	3.71 ± 0.24	0.512	0.002^a^	0.002^a^	0.887
	60d	3.84 ± 0.21	3.78 ± 0.23	4.36 ± 0.15	3.65 ± 0.23	0.672	0.026^a^	0.069	0.238
Urea nitrogen (mg/mL)	0d	1.78 ± 0.03	1.74 ± 0.08	1.79 ± 0.06	1.77 ± 0.04	0.229	0.432	0.924	0.409
	20d	1.96 ± 0.17	1.77 ± 0.22	3.07 ± 0.61	2.09 ± 0.38	0.215	0.037^a^	0.05	0.304
	60d	2.59 ± 0.28	2.55 ± 0.27	3.83 ± 0.66	2.63 ± 0.58	0.898	0.116	0.081	0.766
MCP (mg/mL)	0d	1.30 ± 0.07	1.31 ± 0.04	1.33 ± 0.05	1.33 ± 0.04	0.88	0.486	0.607	0.221
	20d	1.35 ± 0.03	1.37 ± 0.09	2.31 ± 0.48	2.39 ± 0.09	0.613	0.794	0.025^a^	0.001^a^
	60d	1.33 ± 0.02	1.35 ± 0.04	3.04 ± 0.33	3.60 ± 0.26	0.67	0.003^a^	0.002^a^	0.001^a^

^a^Mean significant difference (*P* < 0.05).

### Ruminal fermentation function

The mean ruminal pH of samples from AMP-treated goats ranged from 6.74 to 6.92, which is within the normal physiological range. No significant difference in ruminal pH was observed between AMP-treated groups and control group (*P* > *0.05*; Table 2).

Total volatile fatty acid (T-VFA) and acetate concentrations increased in goats fed AMPs with normal concentrate (significant difference on day 20), but decreased in goats fed double concentrate compared to the control groups (I, III; *P* < *0.05*). The concentrations of ammonia (significant difference with normal concentrate) and urea nitrogen decreased in AMP-treated groups. The acetate + butyrate-to-propionate ratio decreased in AMP-treated groups; however, significant differences were only observed with double concentrate (*P* < *0.05*). The concentrations of microbial protein (MCP, significant difference on day 60 with double concentrate) and propionate (significant difference on day 20 with normal concentrate) increased in AMP-treated groups.

In addition, all indicators (except ruminal pH and urea nitrogen) were elevated in AMP-treated groups compared with groups I and III; the concentrations of T-VFA, acetate, ammonia, and MCP were significantly increased (*P* < *0.05*). Similarly, the concentrations of propionate, butyrate, and the acetate + butyrate-to-propionate ratio were significantly increased on day 20 (*P* < *0.05*) with double concentrate.

### Enzyme activity

Pectinase activity appeared to increase in the AMP-supplemented groups (Table 3), and was higher in AMP-supplemented goats than in the control groups (I, III; *P* < 0.05, except on day 60 with double concentrate). Changes in xylanase, lipase, and amylase activity were associated with concentrate. Xylanase increased with normal concentrate (*P* < 0.05) and decreased with double concentrate; lipase increased with normal concentrate (*P* < 0.05) but did not change with double concentrate; and amylase decreased with normal concentrate (*P* < 0.05) but did not change with double concentrate. No differences in β-glucosidase, carboxymethyl cellulase (CMCase), and protease activity could be detected between AMP-treated and control animals (*P* > 0.05).

**Table 3:** Changes of the activity of enzymes in rumen fluid of goats.

Parameter		Groups				*P-Value*			
		I	II	III	IV	I VS II	III VS IV	I VS III	II VS IV
CMCase (U/mL)	0d	74.39 ± 1.87	73.50 ± 1.50	73.13 ± 1.39	73.20 ± 1.38	0.54	0.79	0.453	0.824
	20d	85.89 ± 2.11	81.03 ± 2.51	89.38 ± 3.21	88.15 ± 1.45	0.15	0.626	0.099	0.016^a^
	60d	112.06 ± 3.33	109.07 ± 3.22	67.97 ± 2.10	71.97 ± 2.43	0.266	0.186	0.001^a^	0.002^a^
Xylanase (U/mL)	0d	10.03 ± 0.33	10.02 ± 0.41	10.24 ± 0.47	10.20 ± 0.35	0.975	0.701	0.424	0.216
	20d	14.56 ± 0.40	18.14 ± 1.27	15.74 ± 1.97	11.27 ± 1.15	0.016^a^	0.019^a^	0.246	0.013^a^
	60d	21.25 ± 0.64	34.57 ± 2.35	26.73 ± 4.34	19.94 ± 1.19	0.001^a^	0.084	0.073	0.005^a^
Pectinase (U /mL)	0d	45.51 ± 3.01	45.15 ± 2.14	45.14 ± 1.65	45.16 ± 1.71	0.728	0.684	0.829	0.992
	20d	37.42 ± 4.56	60.04 ± 1.87	44.23 ± 2.70	47.36 ± 1.34	0.002^a^	0.033^a^	0.013^a^	0.001^a^
	60d	17.19 ± 2.57	26.69 ± 0.53	20.13 ± 2.49	21.01 ± 2.25	0.016^a^	0.433	0.275	0.013^a^
β-glucosidase (U/ mL)	0d	72.62 ± 3.31	72.52 ± 3.23	71.90 ± 2.48	72.05 ± 2.40	0.954	0.75	0.68	0.407
	20d	68.90 ± 4.03	62.40 ± 2.67	60.79 ± 2.69	66.18 ± 3.98	0.168	0.177	0.005^a^	0.006^a^
	60d	59.89 ± 0.49	55.97 ± 2.79	50.82 ± 3.54	59.33 ± 3.61	0.13	0.051	0.016^a^	0.004^a^
Protease (µg /min^.^mL^−1^)	0d	3.25 ± 0.80	3.14 ± 0.35	3.15 ± 0.30	3.17 ± 0.28	0.8	0.571	0.827	0.58
	20d	3.28 ± 0.66	3.18 ± 0.26	2.74 ± 0.62	3.20 ± 0.35	0.775	0.095	0.244	0.957
	60d	4.49 ± 0.43	4.42 ± 0.16	4.16 ± 0.32	4.72 ± 0.48	0.839	0.164	0.471	0.411
Amylase (U/dL)	0d	20.92 ± 0.78	20.89 ± 0.37	20.76 ± 1.17	20.78 ± 0.68	0.962	0.967	0.854	0.778
	20d	24.88 ± 0.33	21.17 ± 1.50	25.59 ± 0.83	25.92 ± 0.61	0.029^a^	0.244	0.25	0.024^a^
	60d	27.62 ± 0.59	25.02 ± 0.58	26.26 ± 1.14	27.71 ± 1.04	0.006^a^	0.163	0.145	0.033^a^
Lipase (U/ L)	0d	19.24 ± 1.69	18.99 ± 1.09	19.92 ± 1.40	19.85 ± 1.38	0.862	0.861	0.316	0.529
	20d	18.81 ± 1.12	23.05 ± 1.36	18.25 ± 2.48	18.76 ± 0.69	0.037^a^	0.634	0.742	0.007^a^
	60d	21.13 ± 2.32	30.50 ± 3.37	32.42 ± 4.18	33.38 ± 3.82	0.041^a^	0.713	0.014^a^	0.468

^a^Mean significant difference (*P* < 0.05).

In addition, β-glucosidase and CMCase (except on day 20) activities appeared to be significantly lower in group III compared to group I (*P* < 0.05); whereas pectinase (except on day 60) and lipase (except on day 20) activities appeared to be significantly higher in group III compared to group I (*P* < 0.05). No differences in xylanase, amylase, and protease activity could be detected between groups I and III (*P* > 0.05).

### Rumen microorganisms

#### Bacterial community structure

Following the removal of low-quality reads from sequencing data, we obtained 1,786,781 total reads for bacteria, with an average of 49,632 reads per sample. The identified bacterial phyla and genera are detailed in Tables 4 and 5 and their respective community compositions are detailed in Supplementary Fig. S1A and B. Bacteroidetes was the dominant bacterial phylum in all goat rumen samples (expect in group III), accounting on average for 40.85% of the bacterial community. The next seven most abundant phyla were Firmicutes, Proteobacteria, Verrucomicrobia, Fibrobacteres, Tenericutes, Spirochaetes, and Cyanobacteria.

**Table 4:** Influence of AMPs on proportion of different bacterial phyla.

Bacterial phylum		Groups				*P-value*			
		I	II	III	IV	I VS II	III VS IV	I VS III	II VS IV
Bacteroidetes	0d	36.00 ± 2.07	36.15 ± 5.15	36.20 ± 4.35	35.93 ± 3.71	0.964	0.638	0.939	0.828
	20d	40.87 ± 2.19	43.68 ± 3.53	34.30 ± 3.67	38.52 ± 2.95	0.483	0.287	0.026^a^	0.105
	60d	47.12 ± 1.10	52.77 ± 4.33	33.57 ± 2.66	35.94 ± 3.72	0.213	0.585	0.023^a^	0.065
Firmicutes	0d	27.02 ± 5.86	28.08 ± 2.58	27.79 ± 4.10	27.57 ± 3.68	0.645	0.735	0.732	0.537
	20d	27.19 ± 1.77	29.65 ± 3.32	35.29 ± 1.53	31.91 ± 1.98	0.387	0.058	0.016^a^	0.474
	60d	18.05 ± 1.07	22.70 ± 1.70	33.72 ± 3.06	26.76 ± 3.56	0.051	0.199	0.022a	0.296
Proteobacteria	0d	19.92 ± 6.46	19.69 ± 4.14	18.99 ± 1.55	18.65 ± 3.53	0.973	0.805	0.859	0.213
	20d	19.23 ± 2.88	7.73 ± 2.46	12.54 ± 2.55	7.20 ± 1.31	0.032^a^	0.042^a^	0.042^a^	0.800
	60d	19.99 ± 0.17	3.29 ± 0.46	14.00 ± 0.33	10.64 ± 0.51	0.001^a^	0.001^a^	0.001a	0.006^a^
Verrucomicrobia	0d	4.60 ± 1.73	5.06 ± 0.38	5.57 ± 1.23	4.58 ± 2.66	0.613	0.557	0.563	0.811
	20d	4.34 ± 0.34	4.45 ± 0.40	6.66 ± 2.20	7.89 ± 0.53	0.760	0.393	0.215	0.005^a^
	60d	2.69 ± 0.35	7.81 ± 2.43	4.82 ± 0.87	8.23 ± 2.02	0.086	0.043^a^	0.034^a^	0.863
Tenericutes	0d	1.75 ± 0.40	2.45 ± 1.23	2.51 ± 0.42	2.60 ± 1.60	0.524	0.936	0.194	0.562
	20d	1.83 ± 0.58	3.72 ± 0.92	1.94 ± 0.50	3.67 ± 1.33	0.094	0.203	0.825	0.970
	60d	2.43 ± 0.44	4.56 ± 0.96	3.12 ± 0.52	5.39 ± 0.71	0.026^a^	0.020^a^	0.317	0.479
Spirochaetes	0d	0.95 ± 0.24	0.69 ± 0.32	0.90 ± 0.25	0.88 ± 0.31	0.499	0.900	0.696	0.428
	20d	1.25 ± 0.17	0.41 ± 0.08	3.02 ± 0.43	1.43 ± 0.40	0.007^a^	0.001^a^	0.023^a^	0.058
	60d	3.00 ± 0.71	1.35 ± 0.21	4.01 ± 0.32	2.73 ± 0.64	0.077	0.101	0.216	0.031^a^
Cyanobacteria	0d	1.67 ± 0.72	1.24 ± 0.44	1.49 ± 0.41	1.48 ± 0.79	0.550	0.999	0.786	0.352
	20d	1.13 ± 0.19	2.48 ± 0.20	1.30 ± 0.07	1.60 ± 0.15	0.003^a^	0.143	0.355	0.004^a^
	60d	0.60 ± 0.11	1.45 ± 0.35	0.77 ± 0.03	1.61 ± 0.55	0.056	0.130	0.076	0.769
Fibrobacteres	0d	5.24 ± 1.00	5.14 ± 0.97	4.68 ± 1.21	5.49 ± 1.10	0.938	0.611	0.696	0.778
	20d	3.93 ± 0.26	5.37 ± 0.18	3.01 ± 0.26	4.25 ± 0.18	0.002^a^	0.002^a^	0.025^a^	0.006^a^
	60d	2.63 ± 0.40	4.36 ± 0.31	2.74 ± 0.21	4.39 ± 0.36	0.008^a^	0.017^a^	0.761	0.463

^a^mean significant difference (*P* < 0.05).

At the phylum level, Proteobacteria appeared to significantly decrease (*P* < 0.05; Table 4) and Fibrobacteres appeared to significantly increase (*P* < 0.05; Table 4) in the AMP-supplemented groups compared with the control groups (I and III). In addition, Bacteroidetes and Proteobacteria appeared to significantly decrease (*P* < 0.05) and Firmicutes appeared to significantly increase (*P* < 0.05) in group III compared with group I.

At the genus level, *Prevotella* dominated the assignable sequences; on average it accounted for 31.35% of total bacteria. *Prevotella* was followed in average relative abundance by *Butyrivibrio* (6.52%), *[Paraprevotellaceae]CF231* (5.02%), *Fibrobacter* (3.75%), *Succinivibrio* (3.04%), and *Anaerovibrio* (1.93%).

*Fibrobacter* and *Anaerovibrio* appeared to increase in the AMP-supplemented groups although a significant increase was only apparent with normal concentrate. *Succiniclasticum* appeared to increase (Table 5), whereas *Succinivibrio*, *Selenomonas*, and *Treponema* appeared to decrease in the AMP-treated groups (Table 5) compared with the control groups (I and III). In addition, *Prevotella*, *Anaerovibrio* (except on day 20), and *Treponema* appeared to significantly increase (*P* < 0.05); whereas *Succinivibrio*, *Selenomonas* (except on day 20), and *Fibrobacter* (except on day 60) appeared to significantly decrease (*P* < 0.05) in group III compared with group I. No differences in *[Paraprevotellaceae]CF231*, *Butyrivibrio*, and *Succiniclasticum* were observed between groups I and III (*P* > 0.05).

**Table 5:** Influence of AMPs on proportion of different bacterial genus.

Bacterial genus		Groups				*P-value*			
		I	II	III	IV	I VS II	III VS IV	I VS III	II VS IV
Undefined genera	0d	39.16 ± 2.73	36.66 ± 1.86	38.09 ± 2.38	38.93 ± 2.07	0.365	0.723	0.158	0.069
	20d	40.27 ± 2.71	39.96 ± 2.76	36.58 ± 3.12	38.41 ± 1.82	0.864	0.280	0.100	0.378
	60d	35.57 ± 1.26	39.34 ± 1.26	35.83 ± 1.46	42.70 ± 1.26	0.081	0.047	0.384	0.126
*Prevotella*	0d	22.20 ± 1.25	22.71 ± 1.55	20.71 ± 1.53	22.73 ± 2.41	0.782	0.466	0.059	0.973
	20d	25.54 ± 2.66	28.71 ± 4.78	31.58 ± 3.90	33.89 ± 3.63	0.467	0.538	0.048^a^	0.349
	60d	27.67 ± 2.54	32.97 ± 6.85	35.82 ± 2.67	35.60 ± 2.95	0.393	0.890	0.029^a^	0.567
*[Paraprevotellaceae]CF231*	0d	7.36 ± 0.52	7.71 ± 1.52	7.63 ± 2.09	7.14 ± 2.95	0.696	0.874	0.870	0.606
	20d	6.03 ± 1.08	5.71 ± 0.81	3.60 ± 1.31	3.26 ± 0.60	0.763	0.687	0.151	0.037^a^
	60d	8.79 ± 1.03	4.72 ± 0.39	4.99 ± 0.21	3.06 ± 0.84	0.119	0.043^a^	0.111	0.130
*Butyrivibrio*	0d	6.51 ± 0.89	6.50 ± 2.03	6.99 ± 0.52	6.71 ± 1.13	0.998	0.719	0.414	0.876
	20d	6.31 ± 0.86	6.52 ± 0.45	7.03 ± 0.73	6.68 ± 0.29	0.555	0.302	0.331	0.685
	60d	6.15 ± 0.07	6.23 ± 0.17	6.54 ± 0.48	6.71 ± 0.22	0.624	0.530	0.323	0.009^a^
*Succinivibrio*	0d	8.23 ± 0.40	7.98 ± 0.54	7.81 ± 0.35	8.37 ± 0.82	0.653	0.413	0.069	0.661
	20d	7.56 ± 0.69	1.00 ± 0.13	4.85 ± 0.45	1.20 ± 0.38	0.003^a^	0.001^a^	0.003^a^	0.375
	60d	3.99 ± 0.52	1.33 ± 0.24	2.28 ± 0.58	2.11 ± 0.94	0.026^a^	0.714	0.040^a^	0.371
*Fibrobacter*	0d	4.60 ± 0.32	4.79 ± 0.68	4.73 ± 0.62	4.61 ± 0.87	0.697	0.885	0.814	0.805
	20d	3.60 ± 0.32	5.20 ± 0.14	2.95 ± 0.25	3.22 ± 0.15	0.005^a^	0.299	0.008^a^	0.004^a^
	60d	2.63 ± 0.40	3.69 ± 0.11	3.07 ± 1.36	3.32 ± 0.45	0.046^a^	0.785	0.678	0.286
*Selenomonas*	0d 0d	3.39 ± 0.44	3.21 ± 1.09	3.27 ± 0.49	3.19 ± 1.37	0.851	0.903	0.813	0.988
	20d	2.95 ± 0.16	1.75 ± 0.45	2.75 ± 0.65	2.99 ± 0.20	0.042^a^	0.625	0.659	0.046^a^
	60d	1.53 ± 0.23	0.57 ± 0.16	0.74 ± 0.22	0.32 ± 0.11	0.025^a^	0.026^a^	0.008^a^	0.095
*Anaerovibrio*	0d	1.92 ± 0.48	2.07 ± 0.24	2.16 ± 0.33	1.96 ± 0.19	0.747	0.159	0.366	0.689
	20d	1.48 ± 0.46	3.35 ± 0.26	1.69 ± 0.36	1.10 ± 0.25	0.046^a^	0.209	0.468	0.012
	60d	1.23 ± 0.27	2.65 ± 0.12	2.22 ± 0.17	2.04 ± 0.20	0.009^a^	0.183	0.049^a^	0.073
*Succiniclasticum*	0d	1.45 ± 0.58	1.57 ± 0.50	1.55 ± 0.51	1.48 ± 0.36	0.753	0.867	0.878	0.542
	20d	1.12 ± 0.09	1.80 ± 0.09	0.85 ± 0.26	1.97 ± 0.14	0.012^a^	0.004^a^	0.289	0.168
	60d	0.04 ± 0.01	1.48 ± 0.36	0.07 ± 0.01	0.53 ± 0.08	0.019^a^	0.016^a^	0.477	0.029^a^
*Treponema*	0d	0.98 ± 0.07	1.15 ± 0.58	1.05 ± 0.35	1.21 ± 0.36	0.701	0.287	0.797	0.883
	20d	1.22 ± 0.15	0.38 ± 0.11	2.35 ± 0.37	1.67 ± 0.24	0.011^a^	0.179	0.012^a^	0.023
	60d	2.95 ± 0.70	1.45 ± 0.15	3.98 ± 0.86	1.73 ± 0.48	0.031^a^	0.043^a^	0.530^a^	0.396

^a^mean significant difference (*P* <0.05).

The Chao1, ACE, Simpson, and Shannon diversity index values of each sample (at the bacterial and ciliate genus level) are shown in Tables 6 and 7, all indices were elevated in the AMP-supplemented groups, especially on day 60. Moreover, all indices were reduced in group III, although these decreases were not statistically significant. These results indicate that AMP supplementation may enhance microbial diversity in the rumen whereas increasing concentrate may reduce it.

**Table 6:** Diversity estimation based on sequence analysis of 16 S rRNA gene libraries of the goat rumen.

erParamet		Bacterial			
		I	II	III	IV
OUT	0d	1221 ± 101	1202 ± 144	1205 ± 153	1239 ± 105
	20d	1211 ± 171.52	1192 ± 168.82	948 ± 172	1058 ± 88
	60d	953 ± 90	1290 ± 111	746 ± 117 ^A^	944 ± 105^B^
Chao1	0d	934 ± 103	948 ± 58	929 ± 54	917 ± 89
	20d	911 ± 167	914 ± 158	676 ± 136	754 ± 61
	60d	713 ± 121	988 ± 103	559 ± 111 ^A^	725 ± 126^B^
ACE	0d	1012 ± 118.40	1023 ± 60.99	1016 ± 152.75	1018 ± 64.38
	20d	1024.85 ± 167.14	1029.74 ± 146.54	750.04 ± 156.02	814.05 ± 113.19
	60d	793.03 ± 106.73	1093.57 ± 106.45	614.01 ± 106.97 ^A^	796.81 ± 120.62^B^
Simpson	0d	0.950 ± 0.049	0.949 ± 0.022	0.951 ± 0.019	0.956 ± 0.036
	20d	0.952 ± 0.050	0.947 ± 0.025	0.957 ± 0.018	0.964 ± 0.025
	60d	0.950 ± 0.044	0.975 ± 0.015	0.939 ± 0.040	0.969 ± 0.014
Shannon	0d	6.560 ± 0.729	6.606 ± 0.516	6.532 ± 0.415	6.599 ± 0.208
	20d	6.650 ± 1.244	6.573 ± 0.687	6.217 ± 0.449	6.562 ± 0.486
	60d	6.228 ± 1.116	7.290 ± 0.335	5.755 ± 0.849	6.663 ± 0.791

^b^The operational taxonomic units (OTUs) were defined with 3% dissimilarity. The diversity indices (Chao1, ACE, Shannon and Simpson) were calculated. ^A,B^Values with different superscripts in the same row differ significantly (*P* < *0.05*)_._

**Table 7:** Diversity estimation based on sequence analysis of 18 S rRNA gene libraries of the goat rumen.

Parameter		Ciliate			
		I	II	III	IV
OUT	0d	116 ± 19	121 ± 8	119 ± 21	124 ± 8
	20d	123 ± 23	130 ± 18	103 ± 15	110 ± 15
	60d	118 ± 19	141 ± 22	108 ± 14	122 ± 7
Chao1	0d	91 ± 13	95 ± 18	89 ± 10	96 ± 12
	20d	98 ± 23	95 ± 18	76 ± 14	85 ± 19
	60d	98 ± 24	116 ± 14	87 ± 14	95 ± 3
ACE	0d	103 ± 24	104 ± 17	105 ± 10	101 ± 21
	20d	106.42 ± 27.99	104.06 ± 20.98	83.82 ± 12.15	99.17 ± 106.42
	60d	107.07 ± 24.21	128.27 ± 21.05	95.50 ± 17.63	108.40 ± 1.72
Simpson	0d	0.764 ± 0.073	0.747 ± 0.046	0.758 ± 0.028	0.765 ± 0.012
	20d	0.766 ± 0.142	0.720 ± 0.128	0.728 ± 0.091	0.769 ± 0.082
	60d	0.784 ± 0.055	0.769 ± 0.071	0.741 ± 0.070	0.811 ± 0.050
Shannon	0d	2.987 ± 0.133	3.019 ± 0.233	3.029 ± 0.058	2.991 ± 0.126
	20d	3.014 ± 0.666	2.819 ± 0.664	2.707 ± 0.593	2.918 ± 0.572
	60d	3.081 ± 0.563	3.074 ± 0.431	2.780 ± 0.311	3.146 ± 0.230

^b^The operational taxonomic units (OTUs) were defined with 3% dissimilarity. The diversity indices (Chao1, ACE, Shannon and Simpson) were calculated.

#### Ciliate community structure

A total of 631,179 quality protozoa sequences were obtained from the 36 samples, with an average of 17,532 reads per rumen sample. Although all animal groups were fed the same diet, there was a high level of variation between individuals in terms of ciliate community composition at the genus level and their respective community compositions are detailed in Fig. S2. The only characteristic in common was the dominant role of *Polyplastron* and *Ophryoscolex* (Table 8).

**Table 8:** Influence of diet and AMPs on proportion of ciliates genera.

Protozoal genus		Groups				*P-value*			
		I	II	III	IV	I VS II	III VS IV	I VS III	II VS IV
*Polyplastron*	0d	40.07 ± 4.64	41.23 ± 4.37	40.21 ± 4.06	42.57 ± 2.07	0.785	0.299	0.977	0.716
	20d	45.37 ± 0.64	33.37 ± 4.71	51.44 ± 7.60	49.09 ± 7.80	0.031^a^	0.783	0.313	0.031^a^
	60d	56.78 ± 4.55	41.28 ± 1.70	65.59 ± 2.93	63.67 ± 2.74	0.013^a^	0.599	0.074	0.003^a^
*Diploplastron*	0d	7.39 ± 1.41	6.80 ± 1.45	7.46 ± 1.27	6.98 ± 0.30	0.719	0.589	0.959	0.846
	20d	6.17 ± 1.04	6.41 ± 0.32	2.60 ± 0.91	2.83 ± 1.71	0.642	0.884	0.086	0.051
	60d	3.31 ± 0.54	3.36 ± 0.37	1.51 ± 0.27	1.81 ± 0.75	0.881	0.581	0.016^a^	0.131
*Entodinium*	0d	4.43 ± 1.05	4.12 ± 0.78	3.67 ± 0.66	4.07 ± 0.21	0.776	0.502	0.207	0.924
	20d	2.65 ± 0.50	0.46 ± 0.16	0.94 ± 0.18	1.01 ± 0.49	0.022^a^	0.726	0.037^a^	0.105
	60d	1.38 ± 0.12	0.60 ± 0.13	1.50 ± 0.21	1.04 ± 0.29	0.002^a^	0.163	0.497	0.165
*Ophryoscolex*	0d	10.86 ± 1.43	11.30 ± 2.98	9.90 ± 4.22	10.31 ± 1.72	0.866	0.886	0.795	0.741
	20d	14.99 ± 7.23	45.07 ± 4.14	24.64 ± 2.60	33.19 ± 4.77	0.006^a^	0.058	0.113	0.014^a^
	60d	27.98 ± 3.44	52.09 ± 2.13	29.09 ± 2.56	31.52 ± 2.07	0.001^a^	0.450	0.641	0.006^a^
*Enoploplastron*	0d	0	0	0	0	—	—	—	—
	20d	0	0	0	0	—	—	—	—
	60d	5.79 ± 1.40	0.16 ± 0.14	0	0	0.023^a^	—	—	—
*Dasytricha*	0d	0.99 ± 0.25	0.79 ± 0.47	1.02 ± 0.29	0.81 ± 0.19	0.626	0.476	0.914	0.936
	20d	0.32 ± 0.40	0.74 ± 0.32	0.99 ± 0.42	0.78 ± 0.54	0.397	0.201	0.261	0.800
	60d	0	0.50 ± 0.42	0.89 ± 0.20	0.93 ± 0.13	0.126	0.829	0.014^a^	0.309
*Isotricha*	0d	36.09 ± 3.74	37.20 ± 3.16	38.02 ± 4.44	36.70 ± 2.92	0.538	0.785	0.427	0.876
	20d	29.87 ± 5.49	13.95 ± 1.36	18.80 ± 5.11	12.89 ± 4.01	0.042^a^	0.362	0.284	0.759
	60d	4.21 ± 0.90	2.01 ± 0.46	1.42 ± 0.31	1.04 ± 0.40	0.038^a^	0.357	0.057	0.007^a^

^a^mean significant difference (*P* < 0.05).

Compared with the control groups (I and III), *Ophryoscolex* appeared to increase in the AMP-supplemented groups (Table 8), although a significant increase was only apparent with normal concentrate. *Polyplastron*, *Entodinium*, and *Isotricha* appeared to decrease in the AMP-supplemented groups, although a significant decrease was only observed with normal concentrate. No differences in *Diploplastron* and *Dasytricha* were detected between AMP-treated goats and control animals (*P* > 0.05). Moreover, no differences in *Polyplastron*, *Ophryoscolex*, and *Isotricha* were evident between groups I and III (*P* > *0.05*).

## Discussion

Microbial community composition in ruminants has previously been linked with animal production traits^21,22^. In the present study, we found that Bacteroidetes was the dominant phylum in all samples (except group III), followed by Firmicutes, Proteobacteria, and Verrucomicrobia. This structure is similar to the rumen bacterial community of sheep inferred from multiplex 454 Titanium pyrosequencing^23^. At the genus level, *Prevotella*, known as an abundant member of the rumen microbiome^24–26^, was the most abundant genus detected, followed by *Butyrivibrio*, *[Paraprevotellaceae]CF231*, *Fibrobacter*, *Succinivibrio*, and *Anaerovibrio*. Many of these genera include organisms that are important cellulose and hemicellulose-degraders; this indicates a rumen bacterial community highly oriented towards fibre degradation. *Polyplastron* and *Ophryoscolex* were the most abundant ciliate genera in this study; the protozoal community composition is similar to that of the A type (dominated by *Polyplastron, Ostracodinium*, *Dasytricha*, and *Entodinium*)^27^. However, many studies have identified *Entodinium* as the predominant protozoal group in ruminants^28–31^. This discrepancy may be due to diet. In this study, forage grass was the main fodder and xylanase and glucanase activities of *Polyplastron* and *Ophryoscolex* are much higher than those of *Entodinium* ^27^. In addition, high-throughput sequencing technology could also affect the true composition of rumen ciliates. Kittelmann *et al*.^32^ reported that smaller-celled genera, such as *Entodinium*, *Charonina*, and *Diplodinium*, tended to be underrepresented, while larger-celled genera, such as *Metadinium*, *Epidinium*, *Eudiplodinium*, *Ostracodinium*, and *Polyplastron*, tended to be overrepresented using the pyrosequencing approach.

Antimicrobial peptides possess broad-spectrum antimicrobial activity and have been used as a new type of feed additive in animal husbandry. A number of recent studies have suggested that dietary supplementation containing an antimicrobial peptide, such as lactoferricin and the lactoferrampin fusion peptide, potato protein, cecropin AD, or antimicrobial peptide P5, reduced the total numbers of aerobes while simultaneously enhancing the total amount of anaerobes and beneficial lactobacilli, thus improving growth performance in weanling pigs^33–36^. In this study, we have shown that dietary supplementation with AMPs improved growth of juvenile goats under two types of concentrate conditions. These results suggest that AMPs can be used to promote growth performance in goats. This is consistent with the finding of Yoon *et al*.^36^ who observed an improvement in the average daily gain and feed efficiency of weanling pigs fed diets supplemented with antimicrobial peptide-A3. Similarly, Jin *et al*.^35,37^ observed an improvement in the average daily gain(ADG) of weanling pigs fed diets supplemented with antimicrobial peptides from *Solanum tuberosum*. Antimicrobial peptides beneficially affect host animals by improving their intestinal balance and creating gut microecological conditions^38–40^. In this study, we found that Proteobacteria were significantly decreased in the AMP-supplemented groups, while Fibrobacteres were significantly increased. This may be due to the fact that Fibrobacteres are anaerobic bacteria^41^, whereas Proteobacteria consist of aerobic bacteria that are mostly pathogenic^42^; the antibacterial peptide could have inhibited the pathogenic bacteria while enhancing the total amount of anaerobes^17^. Dietary supplementation with AMPs has the potential to increase bacterial genera, such as *Fibrobacter*, *Anaerovibrio*, *Succiniclasticum*, and the ciliate genus *Ophryoscolex*, while reducing bacterial genera, including *Selenomonas*, *Succinivibrio*, and *Treponema*, and ciliate genera such as *Polyplastron*, *Entodinium*, and *Isotricha*. However, changes in *Fibrobacter*, *Anaerovibrio*, *Ophryoscolex*, *Polyplastron*, *Entodinium*, and *Isotricha* were related to the amount of concentrate that no significant different in the double concentrate group. Of these, *Fibrobacter* ^43,44^, *Treponema* ^45^, *Ophryoscolex* ^46^, and *Polyplastron* ^47^ are cellulose-degrading microbes and *Succiniclasticum* ^48^, *Entodinium*, and *Isotricha* ^47^ are starch-degrading microbes. *Selenomonas* and *Succinivibrio* degrade both starch and cellulose and *Anaerovibrio* ^49^ are fat-degrading bacteria. Therefore, we hypothesize that the increase in the relative abundance of *Fibrobacter* and *Ophryoscolex* in the normal concentrate group was due to an increase in xylanase and pectinase activities. Similarly, the decrease in the relative abundance of *Isotricha* and *Entodinium* was caused by a decrease in amylase activity in the normal concentrate group; whereas the increase in the relative abundance of *Anaerovibrio* was due to an increase in lipase activity in the same group.

Moreover, the fermentation products of *Fibrobacter*, *Anaerovibrio*, *Treponema*, *Selenomonas*, *O* *phryoscolex*, *Polyplastron*, and *Isotricha* are acetate, propionate, and succinate; the fermentation product of *Succinivibrio* is succinate; and the fermentation products of *Butyrivibrio* are acetate and butyrate. Therefore, an increase in the relative abundance of *Fibrobacter*, *Anaerovibrio*, *Ophryoscolex* in the normal concentrate group may have caused an increase in acetate; whereas a decrease in the relative abundance of *Treponema*, *Selenomonas*, *Polyplastron*, and *Isotricha* in the double concentrate group may have led to the decrease in acetate. Lack of any variation to the relative abundance of *Butyrivibrio* prevented a change in butyrate. Acetate, propionate, and butyrate are the main components in VFAs, accounting for 95% of the total volatile matter content^50^. A change of the acetate + butyrate-to-propionate ratio is related to rumen fermentation mode. Thus, changes in acetate can cause alterations to T-VFA content (increase with normal concentrate and decrease with double concentrate) and the acetate + butyrate-to-propionate ratio (significant decrease with double concentrate). These results indicate that the effects of AMPs on rumen fermentation function and rumen microorganisms in goats were related to the amount of concentrate. It is possible that increased dosage causes similar changes in the double concentrate groups. However, additional studies will be needed to thoroughly elucidate these changes. The alpha diversity indices were elevated in the AMP-supplemented groups in this study, especially on day 60; indicating that AMP supplementation could increase microbial diversity in the rumen.

Previous studies^51,52^ have demonstrated the importance of concentrate supplementation in goat growth and productivity. In this study, we found that the ADG increased with increasing concentrate amount. This result is consistent with the findings of Salim *et al*.^53^ who reported that feeding grazing goats with concentrate supplement may optimize growth performance. The main reason for this may be changes in the rumen bacterial composition of ruminants driven by the amount of dietary concentrate^54^. In the present study, Firmicutes replaced Bacteroidetes as the dominant phylum in group (III) and Proteobacteria were significantly fewer compared to group I. This is in good agreement with data reported by Liu *et al*.^48^ who reported that Firmicutes increased with a high concentrate diet. Similarly, Wetzels *et al*.^55^ observed that Proteobacteria decreased and Firmicutes increased with increasing concentrate doses because of the ability of many Firmicutes to easily degrade fermentable carbohydrates. In terms of bacterial and ciliate genera, *Prevotella* increased with increasing concentrate amount, as reported also by Khafipour *et al*.^56^ and Metzler-Zebeli *et al*.^57^. *Prevotella* is one of the most abundant genera in the rumen of goats because these bacteria possess highly diverse functions, in particular following a high-grain feeding regime. *Anaerovibrio* (on day 60) and *Treponema* increased significantly, whereas *Succinivibrio*, *Fibrobacter* (on day 20), *Selenomonas* (on day 60), and *Diploplastron* (on day 60) decreased significantly with increasing concentrate dosage. Therefore, the higher relative abundance of *Prevotella* and *Anaerovibrio* was due to augmented pectinase and lipase activities, which led to a further increase in T-VFA, ammonia, acetate, and MCP, and ultimately to enhanced goat growth performance.

Moreover, all indices were lower in group III compared to group I, indicating that bacterial diversity depended on dietary concentrate dosage. Similarly, Lillis *et al*.^58^ reported that bacterial diversity was affected to a greater degree by a 90:10 than a 50:50 concentrate:forage ratio.

In summary, this study demonstrates that dietary supplementation with AMPs has beneficial effects on the growth performance, ruminal fermentation function, enzymatic activity, and rumen morphology of juvenile goats; and that these effects are related to concentrate amount. Therefore, AMPs could potentially be used as feed additives for juvenile goats on commercial farms. The detailed mechanism(s) by which AMPs promote growth of juvenile goats and improve their rumen microbial community structure require further clarification.

## Materials and Methods

### Ethics statement

All experimental procedures and animal care performed in the present study were approved according to the recommendations of the Guide of the Sichuan Agricultural University Animal Care and Use Committee (Sichuan Agricultural University, Sichuan, China) under permit NO. DKYB20100805, and all efforts were made to minimize suffering. Field studies did not involve endangered or protected species. Chuanzhong black goats were housed at the experimental farm of the Animal Nutrition Institute of Sichuan Agricultural University.

### Materials

Antimicrobial peptides used were provided by Rota BioEngineering Co., Ltd. (Sichuan, China). AMPs were composed of recombinant swine defensin PBD-mI(DHYICAKKGGTCNFSPCPLFNRIEGTCYSGKAKCCIR) and a fly antibacterial peptide LUC-n(ATCDLLSGTGVKHSACAAHCLLRGNRGGYCNGRAICVCRN) at a blending ratio of 1:1^15^.

### Animal handling

Twenty-four, approximately four-month old, non-castrated Chuanzhong black goats, of average weight (16.17 ± 0.72 kg), were acclimated for 7 days prior to the experiment. All goats were caged and randomly allotted to four dietary treatment groups: I-normal concentrate group (300 g concentrate [per head per day]), II-normal concentrate and antimicrobial peptide group (300 g concentrate + 3.0 g AMPs), III-double concentrate group (600 g concentrate), and IV-double concentrate and antimicrobial peptide group (600 g concentrate + 3.0 g AMPs).

The diet included concentrate (Table 9) and forage (fresh grass). The groups were composed of three replicate pens with 2 goats each, animals were maintained in a house with free access to water, and fed twice daily (at 09:00 and 18:00); the animals maintained their normal herd behaviour.

**Table 9:** Composition and nutrient levels of the concentrate (DM basis).

Ingredients	Content(%)	Nutrient levels	Content(%)
Corn grain	51	DE/(MJ/kg)	13.34
Wheat bran	23	DM	84.27
Rapeseed meal	10	CP	16.66
Rapeseed cake	10	CF	4.17
Fish meal	3	NDF	13.72
NaCl	1	ADF	6.91
Premix^1)^	2		
Total	100		

^1)^Premix provides the following per kg of the diet:Fe(as ferrous sulfate) 30 mg,Cu (as copper sulfate) 10 mg, Zn (as zinc sulfate) 50 mg,Mn (as manganese sulfate) 60 mg,VA 2 937 IU,VD 343 IU,VE 30 IU.

### Sampling and DNA extraction

Rumen fluid samples were collected using a stomach tube on days 0, 20 and 60, prior to morning feeding; the first part of the rumen fluid was discarded to prevent saliva interference. Three goats were selected from each group for sampling(one goats per pen). Rumen pH was measured immediately after collection using a portable pH meter (Model PHB-4, Shanghai Leica Scientific Instrument Co., Ltd., Shanghai, China). Solid feed particles were removed from the rumen fluid by filtration through 4 layers of cheesecloth. Samples were stored at −80 °C for later analysis. Microbial genomic DNA was extracted from rumen samples using a stool DNA kit (OMEGA Bio-Tek, Norcross, GA, USA), according to the manufacturer’s instructions.

### Ruminal fermentation function and enzyme activity analysis

Samples were prepared for VFA analysis and chromatography according to Luo *et al*.^59^. The concentration of NH_3_-N was analysed using visible-light spectrophotometry (Scientific BioMate 3 s, Thermo). NH_4_Cl standards were prepared according to Broderick and Kang^60^. Microbial protein (MCP) in the rumen was analyzed by trichloroacetic acid protein precipitation^61^. The activities of CMCase, xylanase, pectinase and β-glucosidase were measured using commercially available ELISA kits (R&D Systems, Minneapolis, MN, USA). Protease activity was measured as follows: a reaction mixture containing 1 mL casein and 4 mL protease enzyme was incubated for 4 h at 38 °C; at this point, the reaction was stopped by adding 10% trichloroacetic acid. The sample was then centrifuged at 3500 × g for 15 min. Next, 1 mL of supernatant was removed and mixed with 5 mL 0.4 M Na_2_CO_3_ and 1 mL Folin-Ciocalteu’s phenol solution and incubated on the laboratory bench for 15 min. The hydrolysed protein was measured using visual-light spectrophotometry at 680 nm. Concentration and activity of lipase and amylase were measured using commercially available kits (NanJing JianCheng Bioengineering Institute, Nanjing, China).

### Rumen microbial community analysis

The V4 regions of bacterial 16 S rRNA genes and ciliate protozoal 18 S rRNA genes were amplified. Bacterial sequences were amplified using primers 520 F 5′-GCACCTAAYTGGGYDTAAAGNG-3′ and 802 R 5′- TACNVGGGTATCTAATCC-3′; ciliate sequences were amplified using primers V547F 5′-CCAGCASCYGCGGTAATTCC-3′ and V4R 5′-ACTTTCGTTCTTGATYRA-3′. The bacterial amplification mixture consisted of 1 μL (10 μM) of each primer, 1 μL template DNA, 5 μL 5 × reaction buffer, 5 μL 5 × high GC buffer, 0.5 μL 10 mM dNTPs, 0.25 μL Q5 high-fidelity DNA polymerase and 11.25 μL ddH_2_O. The ciliate PCR was carried out in triplicate using 25 μL mixtures containing 1 μL (10 μM) of each primer, 2 μL template DNA, 5 μL 5 × Q5 reaction buffer, 5 μL 5 × Q5 GC high enhancer, 2 μL 2.5 mM dNTPs, and 0.25 μL (5 U/μL) Q5 polymerase. Amplification was performed as follows: initial denaturation at 98 °C for 5 min; 27 cycles of denaturation at 98 °C for 30 s, annealing at 50 °C for 30 s, and elongation at 72 °C for 30 s; plus a final 5-min extension step at 72 °C. PCR products were excised from 2% agarose gels and purified with a QIAquick Gel extraction kit (Qiagen, Venlo, The Netherlands). The remaining DNA was stored at −20 °C until it was used for sequencing. High quality DNA, was sent to Shanghai Paisennuo Biological Technology Co. Ltd for sequencing using an Illumina MiSeqPE250 (Illumina, San Diego, CA, USA).

### Data analysis

Sequence reads were processed and analysed using QIIME pipeline software (version 1.8.0). Chimeric sequences were removed to generate high quality sequences. High-quality sequences were divided and aligned into Operational Taxonomic Units (OTUs) with 97% sequence similarity using the QIIME pipeline software. The highest abundance sequences were compared with template regions in the Greengenes database (Release 13.8, http://greengenes.secondgenome.com/) (bacterial) and NCBI (http://www.ncbi.nlm.nih.gov) database (Ciliate protozoal), and were used to acquire taxonomic information for each OTU and species composition. Alpha diversity indices (including the Simpson index and Shannon index) were obtained using QIIME pipeline software. R software was used to analyze microfloral population structures. The results of these various analyses are expressed as means ± standard error of the mean (SEM). Statistical comparisons were made using paired sample *t* test via a commercially available statistical software package (SPSS 19.0, Business Machines Corporation, Armonk, NY, USA). Differences among treatments were regarded as significant at *P* < *0.05*.

## Electronic supplementary material

Supplementary Information
