# Supplementary material for: Use of antimicrobial peptides as a feed additive for juvenile goats
Source: Sci Rep. 2017 Sep 25;7:12254. doi: 10.1038/s41598-017-12394-4 (PMC5612951; doi:10.1038/s41598-017-12394-4)
Supplement: Supplementary file 1 — Supplementary Information [file 41598_2017_12394_MOESM1_ESM.doc]

# Use of antimicrobial peptides as a feed additive for juvenile goats

Qi Liu^1^**^¶^**, Shuhua Yao^1^**^¶^**, Yun Chen^1^, Shuang Gao^1^, Yanyi Yang^1^, Junliang Deng^1^*, Zhihua Ren^1^, Liuhong Shen^1^, Hengmin Cui^1^, Yanchun Hu^1^, Xiaoping Ma^1^, Shumin Yu^1^


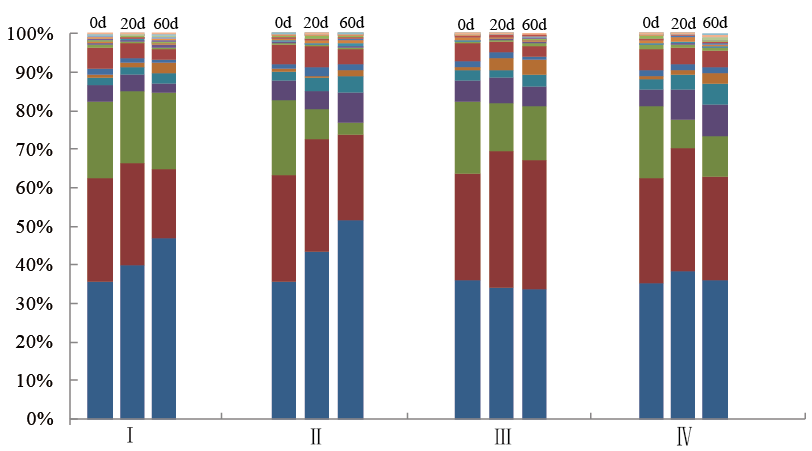


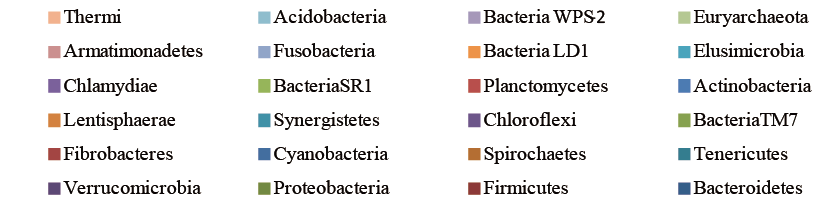


A


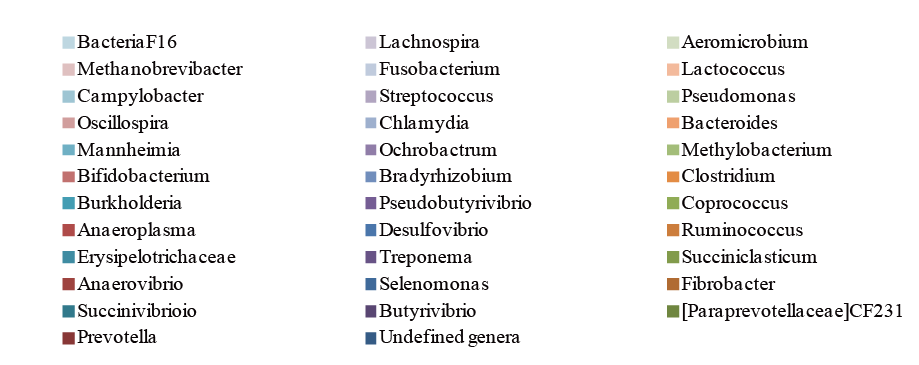

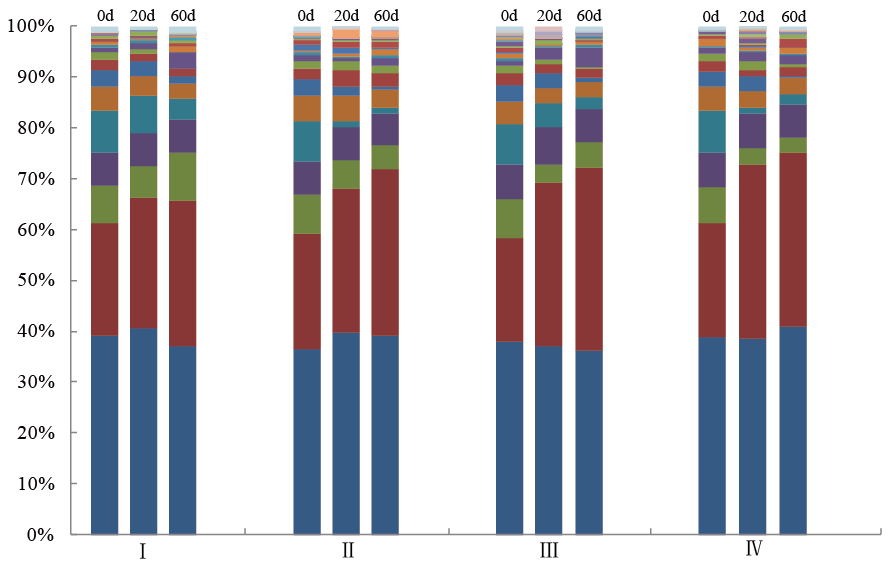


B

Figure S1. Relative abundance of bacterial phyla (A) and genera (B) in rumen samples


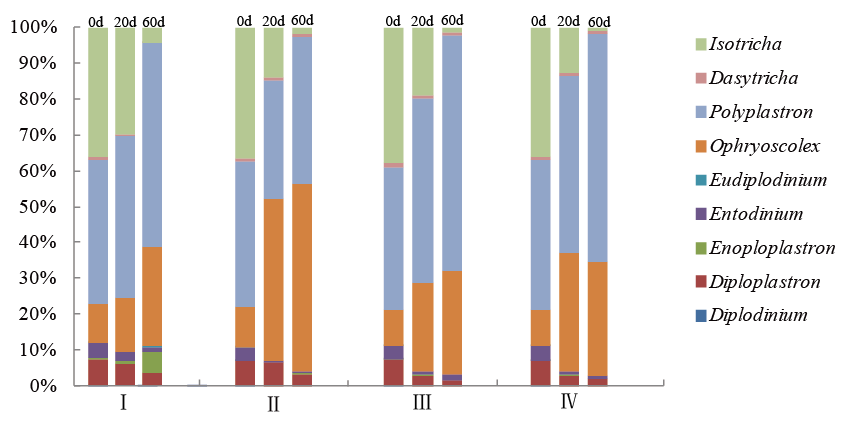


Figure S2 Composition of rumen ciliate at genus level
